# The Study of Ion Transport Parameters in MC-Based Electrolyte Membranes Using EIS and Their Applications for EDLC Devices

**DOI:** 10.3390/membranes12020139

**Published:** 2022-01-24

**Authors:** Shujahadeen B. Aziz, Elham M. A. Dannoun, Rebar T. Abdulwahid, Mohd F. Z. Kadir, Muaffaq M. Nofal, Sameerah I. Al-Saeedi, Ary R. Murad

**Affiliations:** 1 Hameed Majid Advanced Polymeric Materials Research Lab, Physics Department, College of Science, University of Sulaimani, Qlyasan Street, Kurdistan Regional Government, Sulaimani 46001, Iraq; rebar.abdulwahid@univsul.edu.iq; 2 Department of Civil Engineering, College of Engineering, Komar University of Science and Technology, Kurdistan Regional Government, Sulaimani 46001, Iraq; 3 Associate Chair of the Department of Mathematics and Science, Woman Campus, Prince Sultan University, P.O. Box 66833, Riyadh 11586, Saudi Arabia; elhamdannoun1977@gmail.com; 4 Department of Physics, College of Education, University of Sulaimani, Old Campus, Sulaimani 46001, Iraq; 5 Centre for Foundation Studies in Science, University of Malaya, Kuala Lumpur 50603, Malaysia; mfzkadir@um.edu.my; 6 Department of Mathematics and Science, Prince Sultan University, P.O. Box 66833, Riyadh 11586, Saudi Arabia; muaffaqnofal69@gmail.com; 7 Department of Chemistry, College of Science, Princess Nuourah Bint Abdulrahman University, Riyadh 11362, Saudi Arabia; sialsaeedi@pnu.edu.sa; 8 Department of Pharmaceutical Chemistry, College of Medical and Applied Sciences, Charmo University, Chamchamal, Sulaimani 46023, Iraq; ary.murad@charmouniversity.org

**Keywords:** methylcellulose, FTIR, ion transport studies, electrochemical properties, charge-discharge profile, capacitance, electrochemical energy storage device

## Abstract

A solution cast technique was utilized to create a plasticized biopolymer-based electrolyte system. The system was prepared from methylcellulose (MC) polymer as the hosting material and potassium iodide (KI) salt as the ionic source. The electrolyte produced with sufficient conductivity was evaluated in an electrochemical double-layer capacitor (EDLC). Electrolyte systems’ electrical, structural, and electrochemical properties have been examined using various electrochemical and FTIR spectroscopic techniques. From the electrochemical impedance spectroscopy (EIS), a maximum ionic conductivity of 5.14 × 10^−4^ S cm^−1^ for the system with 50% plasticizer was recorded. From the EEC modeling, the ion transport parameters were evaluated. The extent of interaction between the components of the prepared electrolyte was investigated using Fourier transformed infrared spectroscopy (FTIR). For the electrolyte system (MC-KI-glycerol), the *t_ion_* and electrochemical windows were 0.964 and 2.2 V, respectively. Another electrochemical property of electrolytes is transference number measurement (TNM), in which the ion predominantly responsibility was examined in an attempt to track the transport mechanism. The non-Faradaic nature of charge storing was proved from the absence of a redox peak in the cyclic voltammetry profile (CV). Several decisive parameters have been specified, such as specific capacitance (*C_s_*), coulombic efficiency (*η*), energy density (*E_d_*), and power density (*P_d_*) at the first cycle, which were 68 F g^−1^, 67%, 7.88 Wh kg^−1^, and 1360 Wh kg^−1^, respectively. Ultimately, during the 400th cycle, the series resistance *ESR* varied from 70 to 310 ohms.

## 1. Introduction

Solid polymer electrolytes (SPEs) have recently received much interest due to their technical relevance in energy storage devices, including batteries, supercapacitors, fuel cells, and hybrid power sources [1,2,3]. Alkali metal salts are frequently dissolved in polar polymers to produce SPEs [4]. Several characteristics, such as high ionic conductivity, relatively high energy density, absence of leakage, low weight, solvent-free, wide electrochemical windows, and easy handling, make SPEs superior over conventional liquid electrolytes [5]. The potential of an ion-association (ion-pairing) effect has been suggested as one of the primary causes of poor ionic conductivity and concentration polarization. This is owing to the host polymers’ low dielectric permittivity [6]. Without plasticizers, the conductivity of polymer systems is less than 10^−4^ S/cm. Numerous approaches have been presented to address the state-of-the-art issues associated with ion-conducting polymers [7]. Plasticization is among the recent approaches commonly used to overcome the low conductivity issue in the SPEs system using different plasticizers like glycerol and ethylene carbonate. Along with its potential application in electrochemical devices, developing polymeric systems with high ionic conductivity is one of the key aims in polymer electrolyte research [8].

Cellulose is the most common natural organic molecule on the planet, making it a renewable resource and a non-toxic substance in its natural condition [9,10]. Methylcellulose (MC), modified cellulose, is a natural polymer investigated as a viable alternative to synthetic polymers in electrochemical device applications. This is because it is renewable, ecologically benign, plentiful, and affordable [11]. The amorphous polymer MC has a high glass transition temperature (Tg) of 184–200 degrees celsius. The oxygen atoms of MC have lone pair electrons that can act as complexation sites with the salt’s cation [12]. MC has been utilized as an ionic conduction host because of its outstanding thermal, chemical, and mechanical stabilities, excellent film-forming characteristics, and high solubility [13]. MC has excellent film-forming properties, a high mechanical strength, and the ability to produce a clear film. Coatings, pharmaceuticals, and food industry have all used MC [12].

An electrical double-layer capacitor (EDLC) typically consists of two identical carbon-based electrodes. The non-Faradaic process of charge build-up at the double layer on the surface of carbon-based electrodes is a significant limitation in EDLC [14]. This phenomenon needs the large surface area of the electrodes, which is challenging. However, an EDLC possesses several advantages, such as a relatively long lifecycle, high power density, and being lightweight [15]. Moreover, it has been confirmed that the activated carbon and polymer electrolytes used in the capacitor system are compatible [16,17,18]. A moderately large surface area, high electrical conductivity, low-priced cost, and excellent chemical stability are the desired characteristics of activated carbon to be utilized in an EDLC [19]. High-performance, environmentally safe rechargeable batteries are in high demand across the world. Commercially accessible electrochemical devices with high specific capacities have yet to be developed [20]. Polymer electrolytes containing sodium salts have been the subject of little study. When compared to lithium salts, sodium salts have numerous benefits in the production of polymer electrolytes. The softness of sodium-based polymers makes contact with other battery components simpler and more sustainable [21].

The magnesium and sodium-based rechargeable battery systems have recently gained popularity owing to their performance characteristics, which are predicted to be comparable to lithium-based rechargeable batteries. Magnesium is a desirable anode material since it is cost-efficient and safer than lithium due to its natural availability [20,22]. As stated by a recent analysis by Vignarooban et al. [23], sodium-ion batteries are gaining popularity due to their availability and lower cost than lithium-ion batteries (Li). On the other hand, potassium-based salts, such as potassium iodide (KI), have excellent cation mobility through the electrolyte systems, enhancing ionic conductivity. This study reports the successful incorporation of KI salt into the MC host polymer. The impact of glycerol on the electrochemical properties of the system will be explored. Then, the EDLC device performance based on the fabricated polymer electrolyte will be thoroughly investigated.

## 2. Experimental Details 

### 2.1. Materials and Sample Preparation

At ambient temperature, solution-cast solid polymer electrolytes based on MC:KI:glycerol were created. To make a homogenous solution, the MC powder was first dissolved in distilled water as a solvent and then rapidly mixed for 24 h using a magnetic stirrer. Along with constant stirring, the exact quantities of KI (40 wt.%) salt were added to the MC polymer solutions to produce polymer electrolytes. Then, MC-40% KI was glycerolized via various amounts of glycerol ranging from 10 to 50 wt.% in stages of 10%. For MC:KI integrated with 10, 20, 30, 40, and 50 wt.% of glycerol, the samples were classified as MCPN1, MCPN2, MCPN3, MCPN4, and MCPN5. The solution’s components were mixed and then put onto plastic Petri dishes left undisturbed at room temperature to generate a homogeneous solution. Before the EDLC characterization, the electrolytes were kept in a blue silica gel desiccator for subsequent drying. 

### 2.2. Fourier Transform Infrared (FTIR) Spectroscopy 

A Thermo Scientific/Nicolet iS10 FTIR spectrophotometer was used to examine pure MC and MC-doped materials in the 4000–400 cm^−1^ wavenumber range. Each spectrum had a resolution of 2 cm^−1^.

### 2.3. Transference Number Measurement (TNM) and Linear Sweep Voltammetry (LSV)

**Figure 1:**
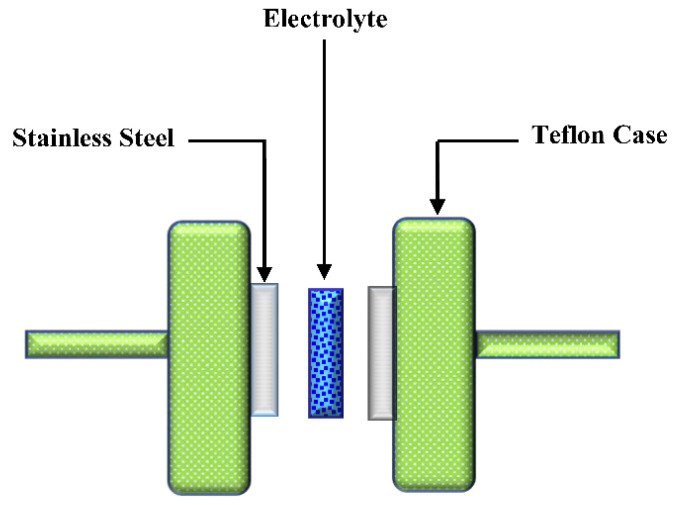
The schematic drawing of the two stainless steel electrodes design for the investigation of transference number measurement (TNM) and linear sweep voltammetry (LSV).

Transference number measurement (TNM) was performed by using a V&A Instrument DP3003 digital DC power supply, in which the ionic (*t_i_*) and electronic (*t_e_*) transference numbers were determined. A Teflon container was used to encapsulate the prepared conducting electrolyte between two identical stainless steel electrodes. At room temperature, the cell perturbation was carried out with the voltage maintained at 0.20 V. On the other hand, by recording linear sweep voltammetry (LSV), a Digi-IVY DY2300 potentiostat was used to investigate the electrolyte electrochemical stability at a scan rate of 100 mV s^−1^. The cell system diagram containing stainless steel electrodes for TNM and LSV measurements is schematically shown in Figure 1.

### 2.4. Electrical Double-Layer Capacitor (EDLC) Preparation

In a planetary ball miller, 0.25 g carbon black and 3.25 g activated carbon were mixed before being added to a solution mixture of 15 mL N-methyl pyrrolidone (NMP) and 0.50 g polyvinylidene fluoride (PVdF). Following that, the final mixture became a thick, black film. 

**Figure 2:**
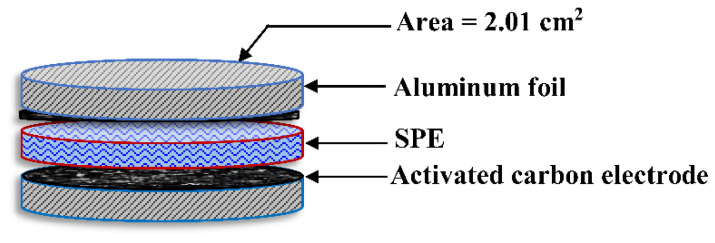
Schematic diagram of the electrical double-layer capacitor (EDLC) cell used in the charge-discharge measurement.

A series of films were formed by cleaning aluminum foil with acetone and flattened over a glass surface. The final solution mixture was placed over the cleaned aluminum foil and then covered using the doctor blade methodology. The films were cut into sliced circulars with a geometric area of 2.01 cm^2^ to produce electrodes. The films in the form of electrodes were dried in the oven at 60 °C. For further drying and to keep them in a dry state, electrode films were then put in a desiccator. To perform the measurements, the most conductive electrolytes (the films in the form of electrodes) were sandwiched between two activated carbon electrodes and put inside a CR2032 coin cell. To learn about the EDLC characteristic, cyclic voltammetry (CV) was obtained at 10 mV s^−1^. The EDLC charge–discharge patterns were examined using a Neware battery cycler with a constant current density of 0.2 mA cm^−2^. Figure 2 depicts a typical EDLC cell construction for charge-discharge applications.

## 3. Result and Discussion 

### 3.1. Fourier Transform Infrared (FTIR) Study

**Figure 3:**
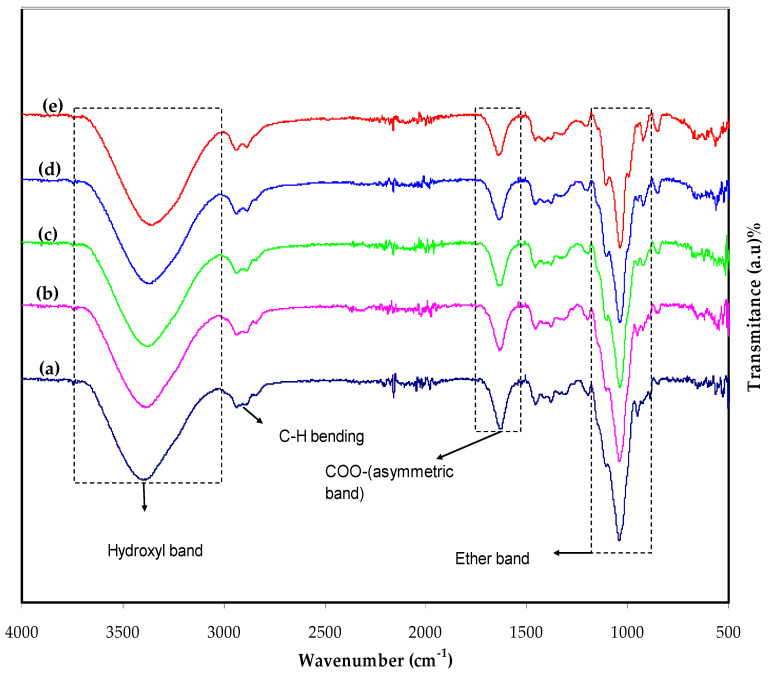
The Fourier transform infrared (FTIR) spectra of the prepared polymer electrolytes (**a**) MCPN1, (**b**) MCPN2, (**c**) MCPN3, (**d**) MCPN4, and (**e**) MCPN5.

The FTIR spectra of MC:KI films containing dissimilar concentrations of glycerol are shown in Figure 3. It is well-known that hydrogen bonding forces the frequencies of stretching vibration of the bonds; therefore, IR spectroscopy can be informative when studying MC-based electrolyte films [24]. In clean MC, the hydroxyl band lies in the wavenumber range of 3447–3458 cm^−1^; however, the range localizes between 1066 cm^−1^ and 1110 cm^−1^ [25]. The stretching vibrations of COO^−^ (symmetric), COO^−^ (asymmetric), C–H (aliphatic), and O–H are attributed to the bands at 1422, 1608, 2931, and 3420 cm^−1^ [26]. O–H stretching at 3458 cm^−1^ and C–H stretching at 2902 cm^−1^ are both absorption bands in pure MC. The C–O stretching from the asymmetric oxygen bridge at 1646 cm^−1^ is between 1065 and 1123 cm^−1^. Interestingly, the C–H bending of MC exhibits distinct small absorption bands in the 1251–1455 cm^−1^ range. Moreover, the C–H vibrations form small bands in the 487–654 cm^−1^ range [27].

In the range of 3650 to 3500 cm^−1^, the unbounded or free hydroxyl group absorbs significantly. The occurrence of a hydrogen-bond hydroxyl group can affect absorption to move to lower frequencies (below 3200 cm^−1^), causing the intensity to rise, the band to expand, and an asymmetrical peak to form. The hydroxyl group in a polymer matrix has an absorption, which is considered an indicator of hydrogen bonding interactions and the strength of hydrogen bonds. In interpreting the spectra of MC films, the hydroxyl stretching at 3466.5 cm^−1^ is critical [24]. In the spectra of MC films, the critical area lies between 3800 and 3000 cm^−1^.

From the difficulties addressed here, the range 950–1250 cm^−1^ was the most instructive. In the IR spectra of cellulose and its ethers, this region features a complicated, strong absorption band of methylcellulose, mainly due to stretching vibrations of C–O bonds [28].

### 3.2. Impedance Analysis

**Figure 4:**
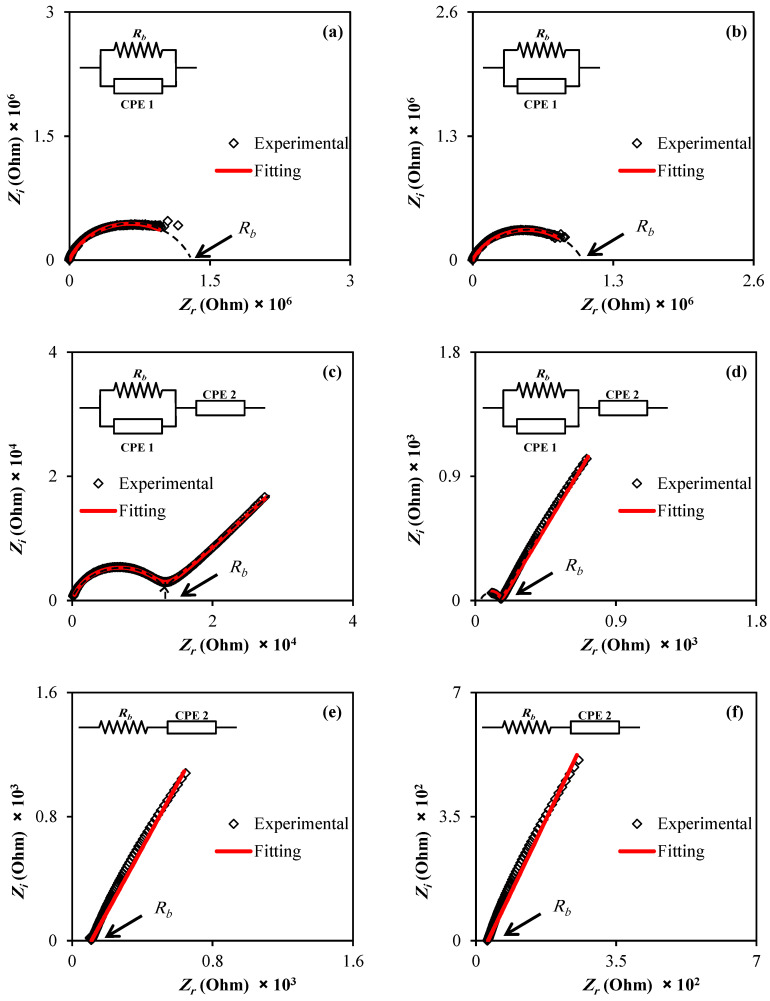
Impedance plots of the (**a**) MC:KI film, (**b**) MCPN1, (**c**) MCPN2, (**d**) MCPN3, (**e**) MCPN4, and (**f**) MCPN5.

The electrical characteristics of electrodes and polymer electrolytes can be studied using electrochemical impedance [29]. Figure 4 shows the electrical impedance graphs (*Z_i_* versus *Z_r_*) for all samples (a–e). From Figure 4a–e, there are two distinct regions: a high-frequency semicircle and a low-frequency spike area. The spike area results from the free charge gathering at the interfacial region, leading to the developing electric double layer (EDL) capacitances [30]. 

In actuality, the complex impedance graphs in the low-frequency area should exhibit a straight line equivalent to the imaginary axis, with an angle of 90°. However, the blocking double-layer capacitance causes this inclination at the blocking electrodes [31,32].

As illustrated in Figure 4, the bulk resistance (*R_b_*) may be calculated using the high-frequency region. It can be seen that two main features are recorded: high-frequency semicircular area and low-frequency spike, as exhibited in Figure 4a–e. The decreasing diameter of the high-frequency semicircle corresponds to increasing salt content and nearly vanishes at 40 wt.% is noticed. It is also noticed that the high-frequency semicircular area is absent in the impedance spectrum, indicating an increase in overall conductivity. This is related to the ion migration at a relatively high salt quantity [33]. It is important to note that the arc is completely absent, making DC conductivity determination virtually impossible (see Figure 4d). DC conductivity was estimated by extrapolating the polarization “spike” in the complex plane to its intersection with the real impedance, as shown in Figure 4d [34]. 

For a variety of experiments, the electrical equivalent circuit (EEC) model was utilised. The EEC is effortless to employ, rapid and gives a comprehensive picture of the whole system. Thus, for impedance spectroscopy analysis, the model was performed [35]. At both high salt quantity and temperatures, the spiketail elongates in the spectra of polymer electrolytes [36]. EEC modeling senses all the circuit components, such as the resistance or capacitance of the samples. The fitting impedance spectra with the EEC model for the chosen samples are shown in Figure 4a–e. As shown, the ideal way to depict the impedance of the MC is to use a parallel combination of resistance and *CPE* (see inset). It is concluded that the films are excellent insulators by showing a relatively high resistivity.

It can be observed that any sample containing 30% glycerol results in a spike and an incomplete semicircle at low and high frequencies, respectively. The connection between *R_b_* and *CPE* can be seen in the high-frequency area, and the *CPE* or the generated double-layer capacitance localizes at the low-frequency zone. In such circumstances, the term *CPE* in the circuit is sometimes used in the ideal capacitor. This is because the SPE behaves differently than an ideal capacitor operating in a pure semicircular pattern; in other words, the SPEs are a pseudo capacitor [37,38].

In addition to the *CPE* capacitor behavior, which explains the depressed semicircle [39,40,41], the low-frequency tail is added as additional *CPE*. At 40 wt.% of plasticizer, only a spike can be seen, confirming the typical diffusion process. In this system, the EEC is represented by a sequence existence of *R_b_* and *CPE* [41,42,43], as demonstrated in an inset in Figure 4d. In the EEC model for MC-KI-glycerol, the impedance of *Z_CPE_* appears in the form of a parallel combination [39,40,41]:

(1) $$Z_{CPE}=\frac{\cos(\pi n/2)}{Y_{m}\omega^{n}}-j\frac{\sin(\pi n/2)}{Y_{m}\omega^{n}}$$

where *Y_m_* is the *CPE* capacitance, *ω* is the angular frequency, and *n* denotes the divergence of the vertical axis of the plot inside the complex impedance plots.

Furthermore, for the equivalent circuit (insets of Figure 4a), the real (*Z_r_*) and imaginary (*Z_i_*) values of complex impedance (Z*) can be stated according to the following mathematical equation [41]:

(2) $$Z_{r}=R_{s}+\frac{R_{1}+R_{1}^{2}Y_{1}\omega^{n1}\cos(\pi n_{1}/2)}{1+2R_{1}Y_{1}\omega^{n_{1}}\cos(\pi n_{1}/2)+R_{1}^{2}Y_{1}^{2}\omega^{2n_{1}}}$$

(3) $$Z_{i}=\frac{R_{1}^{2}Y_{1}\omega^{n_{1}}\sin(\pi n_{1}/2)}{1+2R_{1}Y_{1}\omega^{n_{1}}\cos(\pi n_{1}/2)+R_{1}^{2}Y_{1}^{2}\omega^{2n_{1}}}$$

An incomplete semicircle with a spike (see Figure 4c) can be seen in a Cole-Cole plot with a particular high plasticizer concentration. To match the experimental data points, two constant phase components, one in parallel and the other in series, are essential due to the tail. For the analogous circuit (insets of Figure 4b), the complex impedance (Z*) components, the real (*Z_r_*) and imaginary (*Z_i_*) values can also be written as [39,40]:

(4) $$Z_{r}=R_{s}+\frac{R_{1}+R_{1}^{2}Y_{1}\omega^{n1}\cos(\pi n_{1}/2)}{1+2R_{1}Y_{1}\omega^{n_{1}}\cos(\pi n_{1}/2)+R_{1}^{2}Y_{1}^{2}\omega^{2n_{1}}}+\frac{\cos(\pi n_{2}/2)}{Y_{2}\omega^{n_{2}}}$$

(5) $$Z_{i}=\frac{R_{1}^{2}Y_{1}\omega^{n_{1}}\sin(\pi n_{1}/2)}{1+2R_{1}Y_{1}\omega^{n_{1}}\cos(\pi n_{1}/2)+R_{1}^{2}Y_{1}^{2}\omega^{2n_{1}}}+\frac{\sin(\pi n_{2}/2)}{Y_{2}\omega^{n_{2}}}$$

The semicircle vanishes in the Cole–Cole plot at 40 wt.% of glycerol, as shown in Figure 4d, indicating that the polymer’s resistive component is dominant [40]. The *Z_r_* and *Z_i_* values associated with the EEC, in this case, can be expressed mathematically as follows:

(6) $$Z_{r}=R+\frac{\cos(\pi n/2)}{Y_{m}\omega^{n}}$$

(7) $$Z_{i}=\frac{\sin(\pi n/2)}{Y_{m}\omega^{n}}$$

Various ion transport parameters along with different circuit element parameters are calculated and presented in Table 1 and Table 2. 

### 3.3. Transference Number Measurement (TNM) Study 

In order to employ the polymer electrolyte for application, TNM and LSV must be investigated. A polymer electrolyte’s major charge carrier species must be identified, which can be done through transference number analysis (TNM). The ratio of steady-state current (*I_ss_*) to initial current (*I_i_*) can be used to determine the ion (*t_i_*) and electron (*t_e_*) transference numbers, as illustrated below:

(8) $$t_{i}=\frac{I_{i}-I_{ss}}{I_{i}}$$

**Figure 5:**
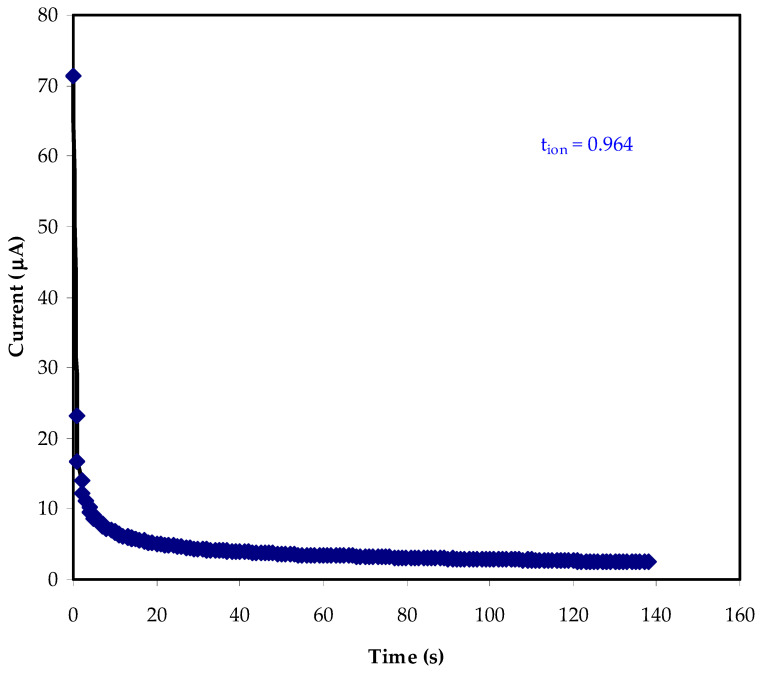
The polarization current against time for the MC-KI-glycerol film with the highest conductivity.

Figure 5 illustrates the polarization curve of current vs. time for the optimal conducting electrolyte. Before reaching a constant value of 71 μA, the current is rapidly reduced.

Because only electrons can flow from side to side the stainless steel electrodes, this speedy decline indicates that the primary charge carrier is ionic rather than electronic. The electrodes that are made of stainless steel impede ion transfer; thus, there is noteworthy decay in current value until it reaches almost constant at 4 μA [44].

The behavior of an ionic conductor is depicted in this phenomenon. The electrolyte’s ionic conductor behavior is indicated by the constant current value [45]. As a result, the electrolyte’s *t_i_* and *t_e_* transference numbers are 0.964 and 0.046, respectively. This demonstrates that the electrolyte’s primary charge carrier is an ion. In contrast, the ionic transference number found in this study is quite comparable to that reported in previous research. Polyvinyl alcohol (PVA)- Magnesium acetate tetrahydrate (Mg(CH_3_COO)_2_) and PVA- magnesium nitrate (Mg(NO_3_)_2_) had ionic transference values of 0.96 and 0.98, respectively, according to Polu and co-workers [46,47].

For Poly(methyl methacrylate) (PMMA): lithium triflate (LiCF_3_SO_3_), Othman, and co-workers [48] reported *t_i_* values ranging from 0.93 to 0.98. (LiCF_3_SO_3_). As a result, the large transference number may be linked to the microscopic parameter’s influence of polymer–ion and ion–ion interactions.

### 3.4. LSV Analysis

**Figure 6:**
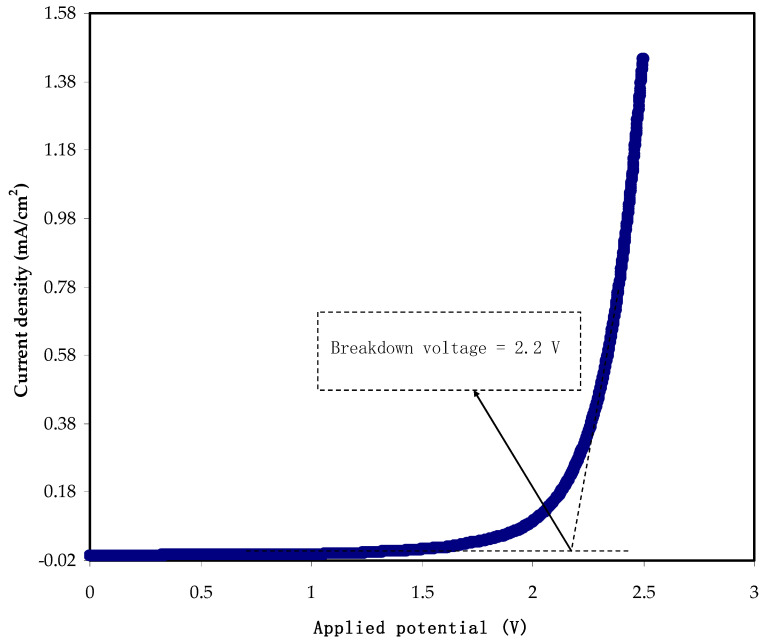
The LSV plot of the MC-KI-glycerol film with the highest conductivity.

One of the requirements of polymer electrolytes to be utilized in energy storage devices is determining electrochemical potential stability. From the response of LSV, one can determine the decomposition potential of the conducting electrolyte, as shown in Figure 6. It can be seen that below 1.5 V, no recording of current, indicating no electrochemical decomposition of the electrolyte before this value of potential.

The potential choice tells us the stop working point of the polymer electrolyte at which the polymer electrolyte will not work at higher than this threshold. In this study, at 2.2 V, the current increase tremendously indicates the impossibility of using electrolytes [49]. Monisha and co-workers [50] state that current passes through the cells at the threshold voltage due to desired electrochemical reactions. In contrast, beyond this, a massive current passes as a consequence of electrolyte breakdown [51]. As reported in the literature, the chitosan-methylcellulose-NH_4_SCN system shows electrochemical stability up to 1.8 V, which is relatively high. In another study, a lithium salt-based biopolymer electrolyte with a decomposition voltage of 2.10 V was recorded by Shukur and co-workers [52]. In addition, the carboxymethylcellulose- ammonium thiocyanate (NH_4_SCN) system has shown electrochemical stability of 1.6 V [53]. In protonic devices, the electrochemical window is widely known to be around 1.0 V [54]. The present results are comparable with that reported for ammonium salt-based polymer electrolytes where a plasticized MC system including NH_4_Br exhibits electrochemical stability up to 1.53 V [55]. Woo and co-workers [56] studied poly (ε-caprolactone) (PCL)-based polymer electrolytes, recording 1.4 V as maximum potential stability.

In proton-based energy devices, the conducting electrolyte of the MC-KI-glycerol system can often be used as an electrode separator. According to the findings of this study, a relatively high conducting electrolyte has the potential stability needed for energy storage device applications.

### 3.5. Cyclic Voltammetry (CV) and EDLC Characteristics

**Figure 7:**
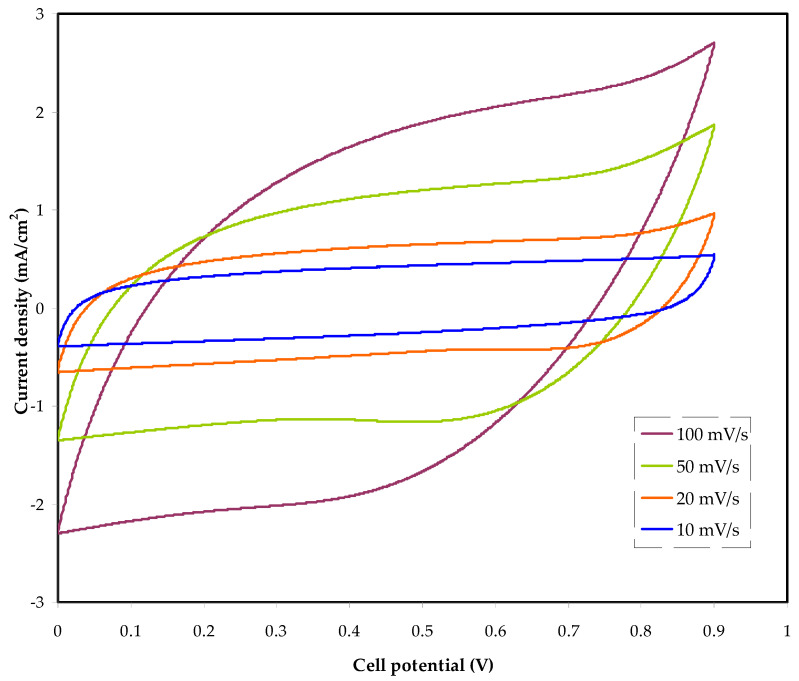
The cyclic voltammetry (CV) for the fabricated EDLC at various scan rates.

Figure 7 shows the cyclic voltammetry (CV) results for the constructed EDLC at various scan speeds. Potentiodynamic electrochemical measurement is used to determine CV. In this analytic approach, the electrode potential is determined linearly in relation to time. The electrode potential can also be shown as a function of time. The cyclic voltammogram trace shows the current versus the applied voltage concerning the working electrode [57]. The CV comeback is a leaf-like form, which is comparatively rectangular. This electrolyte system feature indicates the occurrence of a supercapacitor in EDLC assembly, which is appropriate for utilization.

The lack of a redox peak, which indicates a non-faradaic process in the manufactured supercapacitor and therefore confirms its EDLC behavior [58], confirms that electrons have little involvement. This is owing to activated carbon’s porous structure and internal resistance [59]. Internal resistance and electrode porosity caused the voltage to be current-dependent, making the CV plot less ideal rectangular [60]. 

Additionally, the CV plot shows no redox peak, indicating that the activated carbon electrodes’ surface has a charge double-layer [61]. The following equation can be used to obtain the EDLC’s specific capacitance (*C_spe_*) from the CV plot:

(9) $$C_{spe}=\frac{\int_{V_{1}}^{V_{2}}I\left(V\right)dV}{2m\left(V_{2}-V_{1}\right)\left(\frac{dV}{dt}\right)}$$

The area of the CV plot $\int_{V_{1}}^{V_{2}}I\left(V\right)dV$ produced from the OriginPro 8.5 program is shown below. (*V*_2_ − *V*_1_) is the potential range, and $\frac{dV}{dt}$ is the scan rate [62]. *m* is the activated material mass (activated carbon). The constructed EDLC has a *C_spe_* of 39.32 F g^−1^. The *C_spe_* from the charge-discharge analysis will be compared to this number. The capacitance at different scan rates was determined and is tabulated in Table 3. 

### 3.6. Charge–Discharge Characteristics 

**Figure 8:**
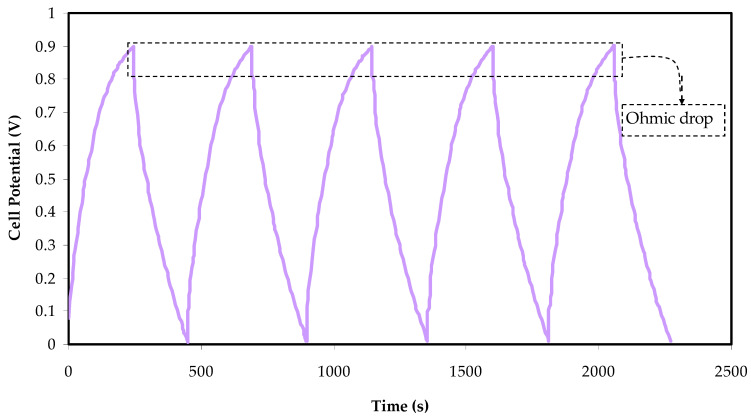
The charge–discharge plot of the fabricated EDLC at 0.5 mA cm^−2^ in the potential range of 0 to 1 V.

The charge–discharge characteristics of the fabricated EDLC are examined using a galvanostatic technique. Figure 8 displays the charge–discharge curve of the manufactured EDLC in the 0 to 1 V potential range at 0.5 mA cm^−2^. 

The discharge slope is approximately linear, showing that the EDLC is capacitive [63]. Once the slope of the discharge curve (s) has been established, the specific capacitance (*C_s_*) can be calculated using the following equation:

(10) $$C_{S}=\frac{i}{sm}$$

**Figure 9:**
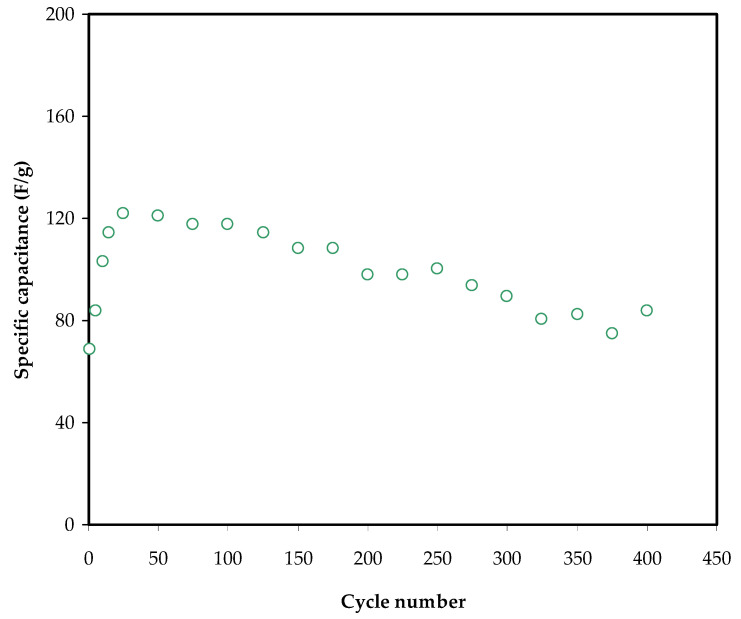
Variation of specific capacitance, *Cs* for the constructed EDLC up to 400 cycles.

The constant current is *i*, and the active material mass is *m*, which is the mass of active carbon. The change of specific capacitance, *C_s_*, for the built EDLC up to 400 cycles is shown in Figure 9. The *C_spe_* rises to 115 F g^−1^ in the fifth cycle and remains constant at 96 F g^−1^ until the 400th cycle.

In the literature, there are two critical records of the specific capacitance values, which are 2.6–3.0 and 1.7–2.1 F g^−1^ corresponding to EDLC cells using Mg- and Li-based Polyethylene glycol (PEO) polymer electrolytes mixed with ionic liquids [64]. Herein, the specific capacitance obtained for the system studied is more significant. Also, the maximum *C_spe_* achieved for the current system is higher than that documented by Mukta Tripathi and SK. Tripathi [65] for an ionic liquid-based gel polymer electrolyte (61.7 F g^−1^). The current work’s specific capacitance is comparable to that reported by Boonen and co-workers [66], which is around 87.3 F/g, and by Łatoszyńska et al. [67], which is about 90 F/g, for gel-based polymer electrolytes.

As a result, polymer blending may be a sole approach to make EDLC with a high specific capacitance at room temperature. The findings of this study might lead to new insights on EDLC manufacturing using natural biopolymers with proton ion conductors. In previous studies, increasing the number of cycles resulted in a significant decrease in *C_spe_*. The development of ion aggregation is thought to be responsible for decreasing these electrochemical characteristics of EDLC.

After the fast charge and discharge cycling, the mobile ions favor being aggregated, resulting in obstructions to ionic transport within the polymer electrolyte. This reduces the amount of ion adsorption at the electrode–electrolyte interface by decreasing ion adsorption at the carbon pores. The EDLC’s specific capacitance, power density, and energy density are all known to decrease as the cycle number rises [68].

The *C_spe_* value found in this study is higher than that published in the literature. Table 4 compares the *C_spe_* of the constructed EDLC to that of earlier studies utilizing various polymer electrolytes. In the table H_3_PO_4_ = orthophosphoric acid, Al_2_SiO_5_ = aluminum silicate, NH_4_NO_3_ = ammonium nitrate, NH_4_C_2_H_3_O_2_ = ammonium acetate, LiTf = lithium triflate, EMITf = 1-ethyl-3-methylimidazolium trifluoromethanesulfonate.

The equivalent series represents the internal resistance of the EDLC and can be found from the resistance *R_esr_*. From the following relationship, it is possible to determine the EDLC’s *R_esr_*:

(11) $$R_{esr}=\frac{V_{d}}{i_{}}$$

A remarkable voltage drop (*V_d_*) before each discharging operation can be seen in Figure 8. The voltage drop lies between 0.04 to 0.12 V, caused by internal resistance within the EDLC. Three different resistances within EDLC can be counted: the current collector, the bulk of the electrolyte, and the interfacial area between the electrodes and electrolyte [72,73,74].

**Figure 10:**
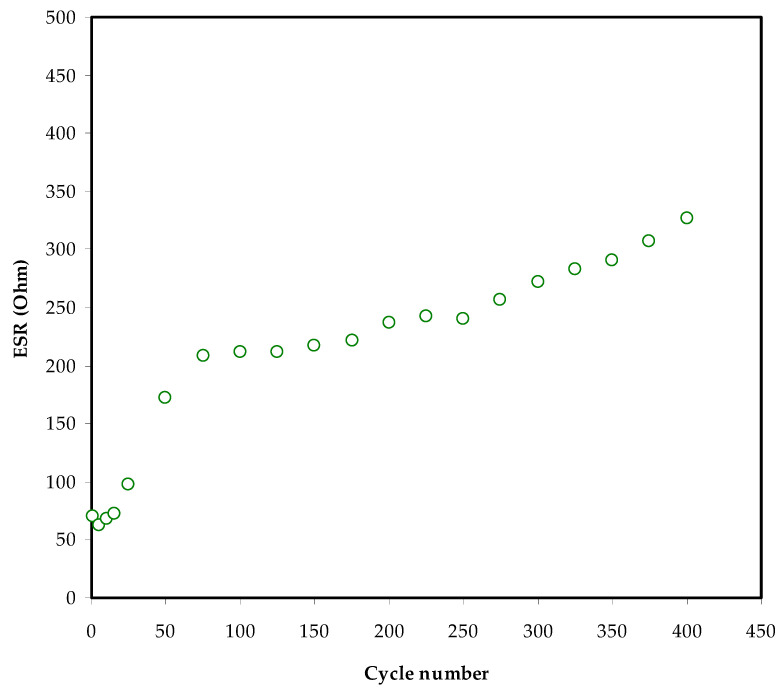
The *R_esr_* of the EDLC for 400 cycles.

The *R_esr_* of the EDLC at 400 cycles is exhibited in Figure 10. The *R_esr_* varies from 70 to 310 Ω. It is worth noting that the weakening of the solid polymer electrolytes in the EDLC investigated by Kumar and Bhat [74] occurs owing to the boost in voltage drop during the charge–discharge cycle, which leads to an increase in *ESR*. When *ESR* is taken into account, the ionic liquid integrated with poly (ethylene oxide) (PEO)-based polymer electrolyte has a value of 1300 Ω [64], while the existing electrolyte has a value that is relatively low. Asmara et al. [75] recorded a low *R_esr_* value for compatible electrode–electrolyte contact, implying that ions move quickly from the electrolyte to the electrode surface, creating an electrical double-layer.

**Figure 11:**
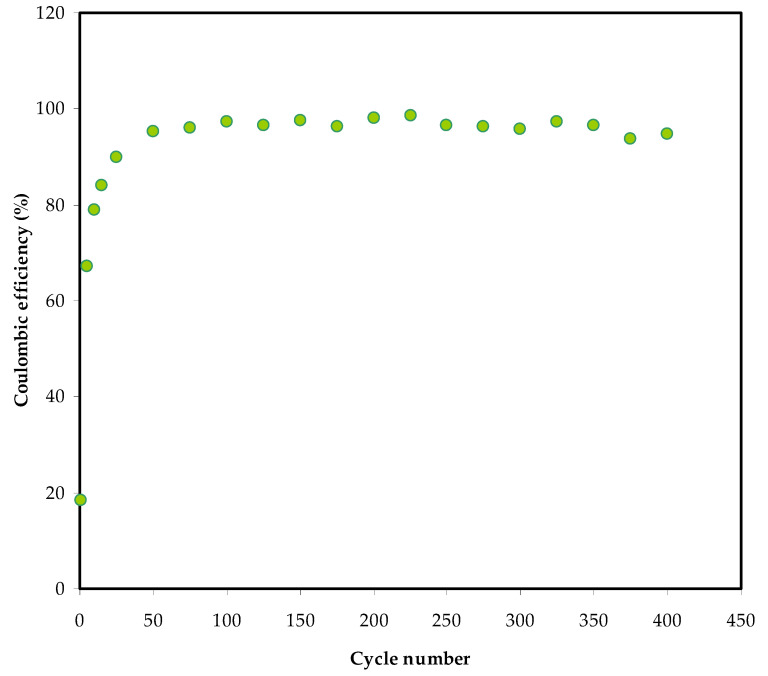
The *η* of the EDLC up to 400 cycles.

Another significant metric for the EDLC’s cycle stability is coulombic efficiency (*η*, and it can be computed using the following formula:

(12) $$\eta =\frac{t_{d}}{t_{c}}\times 100$$

where *t_d_* and *t_c_* stand for discharge and charge times, respectively. Figure 11 depicts the *η of* the EDLC after 400 cycles. At the first cycle, the coulombic efficiency *η* is 67%, increasing to 93% and 96% at the 50th and 100th cycles, respectively. At the 200th cycle, it is 97%, then drops to 95% and stays there until the 400th cycle. The EDLC is thought to have credible electrode-electrolyte contact since the *η* is more than 90% [76,77]. An earlier study has recorded 96.1% for an EDLC device over 300 cycles composed of a PVA host doped with 40 wt.% potassium iodide (KI) and 40 wt.% glycerol [38]. In another work, the fabricated ELDC device with polymer-based electrolyte consisted of chitosan-magnesium acetate Mg(CH_3_COO)_2_ and reached an average efficiency of 96.1% up to 1000 cycles [51]. 

**Figure 12:**
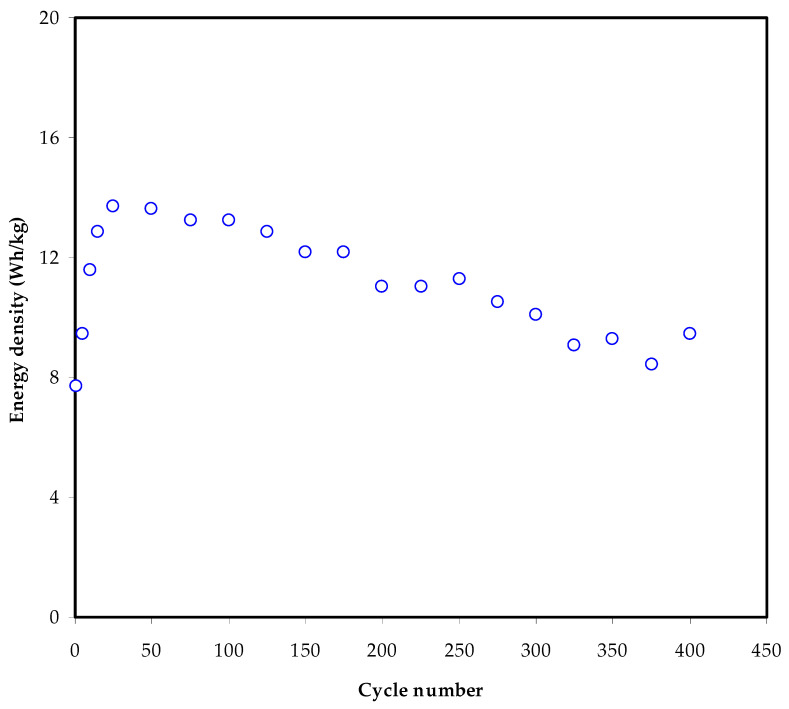
The energy density (*E_d_*) for the assembled EDLC throughout 400 cycles.

The manufactured EDLC’s energy density (*E_d_*) may be determined using the equation:

(13) $$E_{d}=\frac{C_{s}V}{2}$$

where *V* equals 1 V in this case. Figure 12 illustrates the constructed EDLC’s energy density (*E_d_*) across 400 cycles. *E_d_* is 7.88 Wh kg^−1^ in the first cycle, as seen in the diagram. From the 200th cycle, the *E_d_* rises to 13.81 Wh kg^−1^ and subsequently declines to 11 Wh kg^−1^.

It can be observed that the energy barrier for ion transport is almost constant from the 10th to the 100th cycles [78]. Mukta Tripathi and SK. Tripathi reported an energy density of 11 Wh Kg^−1^ for the current EDLC assembly, which is significantly greater than that reported for ionic liquid-based gel electrolytes [65]. Furthermore, compared to the earlier study, the present energy density was significantly higher than that for chitosan:dextran (Dex) studies (1.4 Wh kg^−1^ and 0.86 Wh kg^−1^) using NH_4_F and LiClO_4_ salts, respectively [79,80]. Unfortunately, it is less than the value reported (8.63 Wh kg^−1^) for biopolymer mix electrolytes containing NH_4_SCN [81]. Furthermore, an average of 7 Wh/kg energy density for plasticized CS-LiClO_4_ system is achieved over 300 cycles [18]. 

**Figure 13:**
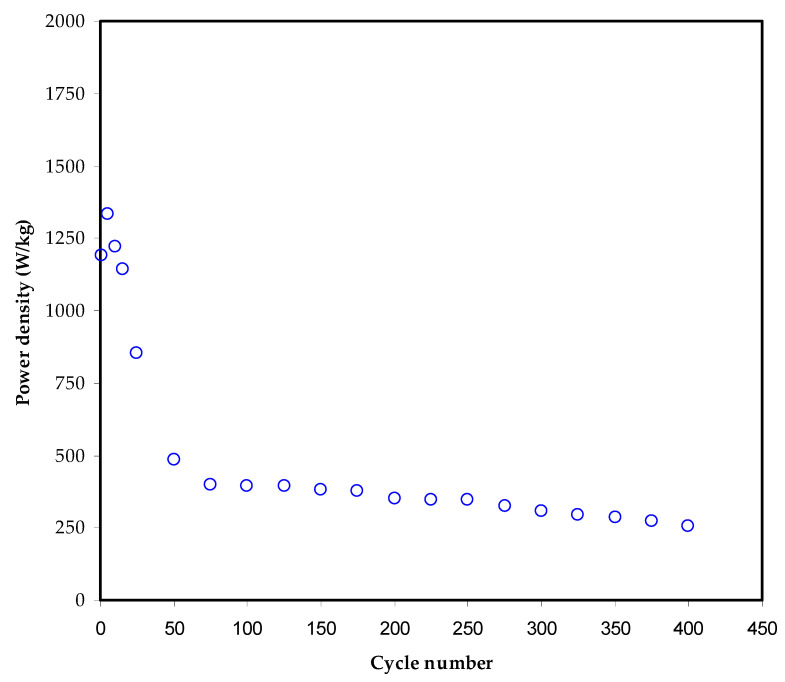
Power density (*P_d_*) for the assembled EDLC throughout 400 cycles.

Figure 13 shows the power density (*P_d_*) for the constructed EDLC over 400 cycles, as computed using the equation:

(14) $$P_{d}=\frac{V^{2}}{4mR_{esr}}$$

The *P_d_* of 1360 W kg^−1,^ and 500 W kg^−1^ were recorded at the 1st and 50th cycles, respectively. From the 50th to the end of the cycles (400th cycle), the value of *P_d_* is 302 to 370 W kg^−1^, respectively. An average of 340 W kg^−1^ has been recorded over the whole cycling process. A comparable trend of *R_esr_* and power density patterns of the EDLC have been obtained, as shown in Figure 13. Coromina and co-workers [82] demonstrated that the energy stored in EDLCs could be supplied via aqueous H_2_SO_4_ or ionic liquid electrolytes, achieving a power density of more than 1 kW kg^−1^, bridging the gap between EDLCs and batteries. Moreover, Hadi et al. documented a 300 W/kg power density over 400 cycles for chitosan host doped with ammonium iodide and Zn(II)-complex and plasticized with glycerol [19]. According to the current study results, it is possible to construct EDLC cells with a high power density using biopolymer-based mix electrolytes. It has been established that energy and power densities are directly related to active material mass loading and other parameters.

Energy and power density are inversely proportional to mass loading. Therefore, low mass loading and low current always result in improved electrochemical performance, according to reference [83].

Table 5 provides the EDLC device performance for various MC polymer-based electrolyte systems. The present work and those published previously highlights the fact that biopolymers are significant for polymer electrolyte preparation and device fabrication. The necessity for bendy energy storage devices including EDLCs and batteries for new technologies is encouraging researchers to invent and discover new materials. Of course, problems in this field are that devices based on biopolymers are not stable for higher cycle numbers. From the above analysis of energy and power densities of current EDLC devices and comparison to previously fabricated devices (see Table 5), it can be concluded that some critical factors can contribute in achieving better device performance. First, selection of the host polymer matrix is among the critical components and plays the key role in getting good device performance. In EDLC application the host polymer should provide good ionic conduction along with suitable chemical, thermal and mechanical stability. These properties give the fabricated EDLC device good outputs and long cyclability. Second, choosing a suitable dopant salt is another important constituent that need to be taken into consideration during employment of the polymer electrolyte in electrochemical device applications. Salts with low lattice energy can easily undergo the dissociation process, while large lattice energy salts cannot be dissociated simply by the host medium which also creates a free carrier issue. Moreover, using plasticizer can improve ionic conduction, however, the inclusion of large amount can cause mechanical weakness and increase reaction toward the electrodes. From the above discussion it can be concluded that finding suitable polymer-based electrolytes that lead to both high energy and power density along the range cycle is quite challenging and needs thorough scientific exploration. Our research group works to generate polymer electrolyte films with high conductivity and high-quality device performance. To the best of our knowledge, all obtained results based on biopolymers should be presented to the scientific community in order to shed light on the progress in this field. The achieved results promise to produce devices with high performances in the future; but of course, this needs more attention of scientists. The devices based on biopolymers are crucial to be focused because they are totally non-toxic and avoid poisonous materials. As mentioned above, numerous factors including functional groups on the polymer chain backbone, lattice energy of salt and plasticizers affect the performance of the devices. Thus, different polymers, salts or plasticizers should be examined and their results must be presented for the scientific community in the form of research articles. Nonetheless, the achieved EDLC device performance based on biopolymers so far has been promising, and higher performance in the near future is expected.

## 4. Conclusions

A biopolymer electrolyte based on MC:KI with various quantities of glycerol plasticizer was produced for EDLC device applications. FTIR spectra were used to validate the interactions and complexation between the electrolyte components. The EIS data showed that when the glycerol content grew from 10% to 50%, the resistance to charge transfer at the bulk of the electrolyte reduced due to an increase in charge carrier density. EEC models were utilized to determine the ion transport parameters such as carrier density, diffusion and mobility. The electrolyte containing 40 wt.% glycerol exhibited the highest conductivity, measuring 5.14 × 10^−4^ S/cm. In TNM experiments, the (*t_ion_*) was determined to be 0.964, suggesting that ions were the primary charge carrier. The most conducting sample exhibited an electrochemical stability window of up to 2.2 V, validating the electrolyte’s suitability for the EDLC, according to the LSV research. The CV response had no discernible redox peak, indicating capacitance behavior. *C_s_*, *η*, *E_d_*, and *P_d_* were discovered to have starting values of 68 F/g, 67%, 7.88 Wh/kg, and 1360 Wh/kg, respectively.

## Figures and Tables

**Table 1:** Ion transport parameters of different plasticized systems.

Sample	σ_dc_ (S/cm)	D	μ	*n*
MC:KI	1.19 × 10^−8^	-	-	-
MCPN1	1.54 × 10^−8^	-	-	-
MCPN2	1.30 × 10^−6^	2.27 × 10^−11^	8.85 × 10^−10^	9.14 × 10^21^
MCPN3	9.35 × 10^−5^	1.14 × 10^−11^	4.44 × 10^−10^	1.31 × 10^24^
MCPN4	1.40 × 10^−4^	2.31 × 10^−6^	8.99 × 10^−5^	9.73 × 10^18^
MCPN5	5.14 × 10^−4^	1.79 × 10^−6^	6.97 × 10^−5^	4.61 × 10^19^

**Table 2:** Calculated different circuit parameters for the prepared polymer electrolytes.

Sample	p1	p2	*CPE*1	*CPE*2
MC:KI	0.76	-	2.86 × 10^−9^	-
MCPN1	0.72	-	3.70 × 10^−9^	-
MCPN2	0.87	0.52	8.33 × 10^−10^	1.57 × 10^−6^
MCPN3	0.86	0.69	3.33 × 10^−9^	7.69 × 10^−6^
MCPN4	-	0.44	-	8.33 × 10^−6^
MCPN5	-	0.39	-	1.45 × 10^−5^

**Table 3:** Specific capacitance (*C_s_*) of the EDLCs using CV curves.

Scan Rate	V2 − V1	Capacitance
0.1	0.9	16.42
0.05	0.9	23.23
0.02	0.9	31.32
0.01	0.9	39.32

**Table 4:** Proton-based EDLC studies with their relative specific capacitance value.

SPE System	*C_s_* (F/g)	Cycles	Reference
Chitosan-H_3_PO_4_-Al_2_SiO_5_	0.22	100	[69]
Chitosan-H_3_PO_4_-NH_4_NO_3_-Al_2_SiO_5_	0.25	100	[69]
PVA-NH_4_C_2_H_3_O_2_	0.14	Not stated	[70]
MC-NH_4_NO_3_	1.67	100	[71]
PEO-LiTf-EMITf	1.70	Not stated	[64]
MC-KI-Glycerol	96	400	This work

**Table 5:** Polymer-based electrolyte EDLC device performance.

SPE System	Energy Density (Wh kg^−1^)	Power Density (W kg^−1^)	Cycle Number	Ref.
MC–PEG–NH_4_NO_3_	3.9	140	4	[71]
PS-MC–NH_4_NO_3_-glycerol	2.3	385	1000	[62]
CS-MC-NH_4_I-glycerol	0.77	578	100	[84]
MC-Dex-NH_4_I	6.3	170	100	[85]
CS-MC-NH_4_SCN	8.63	555	100	[81]
CS:MC:NH4I	-	-	100	[61]
MC-KI-Glycerol	11	340	400	This work

NH_4_I = ammonium iodide, PEG = polyethylene glycol, PS= potato starch.
